# Development and prognostic evaluation of a combined SII–LNR score in resectable gastric and gastroesophageal junction adenocarcinoma treated with perioperative FLOT: a retrospective single-center study

**DOI:** 10.7717/peerj.21499

**Published:** 2026-06-29

**Authors:** Mert Tohumcuoğlu, Mahmut Büyükşimşek

**Affiliations:** Department of Medical Oncology, University of Health Sciences, Adana City Training and Research Hospital, Adana, Turkey

**Keywords:** Gastric adenocarcinoma, Overall survival, Disease-free survival, Lymph node ratio, Systemic immune-inflammation index, Gastroesophageal junction adenocarcinoma, FLOT

## Abstract

**Background:**

The systemic immune-inflammation index (SII) and lymph node ratio (LNR) have both been associated with survival in gastric and gastroesophageal junction (GEJ) adenocarcinoma, but their combined prognostic value is unclear in patients treated with perioperative fluorouracil, leucovorin, oxaliplatin, and docetaxel (FLOT). We developed a combined SII–LNR risk score and assessed its association with overall survival (OS) and disease-free survival (DFS).

**Methods:**

This retrospective single-center cohort included 153 patients with resectable gastric or GEJ adenocarcinoma treated with perioperative FLOT. SII was calculated from pretreatment complete blood counts and LNR from postoperative pathology. OS and DFS were evaluated using Kaplan–Meier methods and Cox proportional hazards regression. Cut-offs were defined using receiver operating characteristic (ROC) analysis with the Youden index, and area under the curve (AUC) values were reported.

**Results:**

ROC-derived cut-offs were 715 for SII (AUC 0.602) and 0.13 for LNR (AUC 0.751). The combined SII–LNR score categorized patients into low-risk (*n* = 50, 32.7%), moderate-risk (*n* = 67, 43.8%), and high-risk (*n* = 36, 23.5%) groups. Median OS was 41.5, 30.5, and 17.2 months, with 5-year OS rates of 40.5%, 17.1%, and 4.4%. Median DFS was 32.4, 15.6, and 10.8 months, with 5-year DFS rates of 34.2%, 17.4%, and 5.7%. Compared with the low-risk group, the risk of death was higher in the moderate- and high-risk groups (HR 1.93, 95% CI [1.15–3.26]; HR 4.13, 95% CI [2.37–7.22]) and the risk of recurrence or death was also higher (HR 2.07, 95% CI [1.25–3.42]; HR 3.58, 95% CI [2.12–6.02]).

**Conclusion:**

The combined SII–LNR score stratified patients with resectable gastric and GEJ adenocarcinoma treated with perioperative FLOT into distinct postoperative OS and DFS risk groups. If confirmed in multicenter cohorts, it may help identify patients who warrant closer postoperative follow-up.

## Introduction

Gastric cancer remains one of the leading causes of cancer-related death worldwide. Because early-stage disease is frequently asymptomatic or nonspecific, many patients are diagnosed with locally advanced disease (Joshi & Badgwell, 2021; Repetto et al., 2023). Perioperative chemotherapy has been shown to improve outcomes compared with surgery alone in resectable locally advanced gastric and gastroesophageal junction (GEJ) adenocarcinoma (Li et al., 2022). Among perioperative regimens, fluorouracil, leucovorin, oxaliplatin, and docetaxel (FLOT) demonstrated superior survival and pathological response compared with older epirubicin-based triplet regimens in the FLOT4-AIO trial (Al-Batran et al., 2019). More recently, perioperative chemoimmunotherapy strategies have also entered the treatment landscape; in the phase 3 MATTERHORN trial, perioperative durvalumab plus FLOT improved event-free survival compared with placebo plus FLOT in resectable gastric or GEJ adenocarcinoma (Janjigian et al., 2025).

Despite advances in perioperative treatment, survival outcomes remain heterogeneous among patients receiving the same regimen. This heterogeneity highlights the need for simple and accessible postoperative prognostic tools that can support risk stratification after resection (Wang et al., 2021).

Inflammatory markers derived from routine blood tests have attracted interest because of the established relationship between systemic inflammation and cancer progression (Zhang et al., 2020; Ma et al., 2023; Golder et al., 2021; Rassouli et al., 2015; Yodying et al., 2016). The systemic immune-inflammation index (SII), calculated from neutrophil, lymphocyte, and platelet counts, reflects the balance between tumor-promoting inflammatory activity and host antitumor immune response. Previous studies have shown that SII has prognostic value in several solid tumors, including gastric cancer (Hirahara et al., 2021; Wang & Zhu, 2019; Nakamoto et al., 2023; Zheng et al., 2023; Ji & Wang, 2020).

Likewise, the lymph node ratio (LNR), defined as the ratio of metastatic to examined lymph nodes, may provide prognostic information beyond conventional nodal staging, especially after neoadjuvant treatment (Marchet et al., 2007; Zhu et al., 2021; Jiang et al., 2022).

Pretreatment SII and postoperative LNR may capture distinct yet complementary prognostic information related to systemic inflammatory response and nodal tumor burden. Therefore, evaluating these parameters together may improve risk stratification in resected gastric and GEJ adenocarcinoma. However, their clinical timing differs: SII is available before therapy, whereas LNR is determined only after surgery. Thus, the combined SII–LNR score should be interpreted primarily as a postoperative prognostic tool rather than a pretreatment predictor of response to perioperative therapy.

To our knowledge, this combined strategy has not been specifically investigated in patients with resected gastric and GEJ adenocarcinoma treated with perioperative FLOT. Accordingly, we aimed to evaluate the prognostic value of pretreatment SII and postoperative LNR, both individually and in combination, for overall survival (OS) and disease-free survival (DFS) in this setting.

## Materials and Methods

### Study design and site

This retrospective cohort study was conducted at the Department of Medical Oncology, Adana City Training and Research Hospital, Adana, Turkey.

### Patient selection and study population

**Figure 1:**
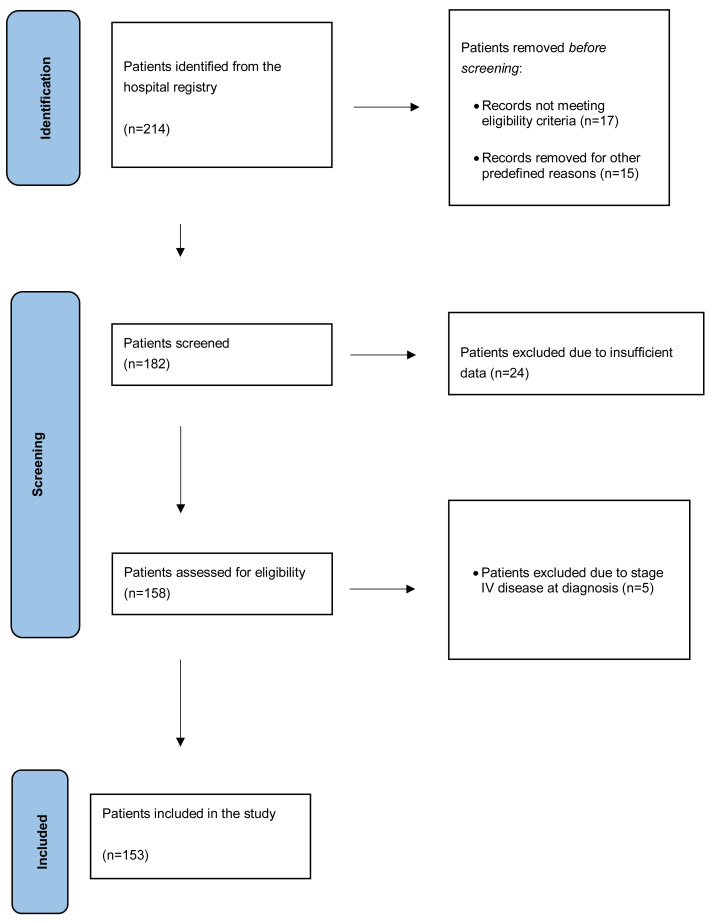
Flowchart of patient selection from the hospital registry.

This retrospective single-center cohort study included patients with resectable gastric or gastroesophageal junction (GEJ) adenocarcinoma diagnosed between January 2019 and October 2024 who received perioperative FLOT at the Medical Oncology Department of Adana City Training and Research Hospital. Follow-up was updated through 14 August 2025. A total of 214 patients with gastric or GEJ adenocarcinoma who received perioperative FLOT during the study period were retrospectively identified from the institutional electronic health record system. After initial screening, 17 patients who did not meet the eligibility criteria and 15 patients excluded for other predefined reasons were removed. Of the remaining 182 patients assessed for eligibility, 24 were excluded because of missing or incomplete clinical, laboratory, or pathological data, and five were excluded because distant metastasis was documented during detailed eligibility review. The final analytic cohort consisted of 153 patients (Fig. 1).

#### Inclusion criteria

(I) Age ≥18 years

(II) Histopathological diagnosis of gastric or GEJ adenocarcinoma

(III) No distant metastasis at diagnosis

(IV) Receipt of surgery and perioperative FLOT.

#### Exclusion criteria

(I) Organ failure

(II) Concomitant primary malignancy other than non-melanoma skin cancer

(III) Documented autoimmune disease at baseline, which could confound SII

(IV) Distant metastasis or clinical T1 disease

(V) Failure to undergo surgery or failure to complete perioperative FLOT

(VI) Missing or incomplete required clinical, laboratory, and pathological data

### Clinical and demographic data collection

Clinical and laboratory data, including demographics, tumor location, complete blood counts, liver and kidney function test results, histopathological subtypes, and clinical and pathological stages, as well as the dates of treatment initiation, surgery, recurrence, and death, were retrieved from the institutional electronic health record system and patient files and recorded for each patient. According to the institutional clinical and pathological records, 118 patients had gastric adenocarcinoma and 35 had GEJ adenocarcinoma. In the retrospective dataset, tumor location was additionally recorded as upper, middle, or lower third. Therefore, the upper-third category included both proximal gastric tumors and GEJ tumors. Detailed Siewert subclassification was not consistently available in the retrospective dataset and was not included in the analyses.

### Histopathological assessment

Histopathological variables collected from pathology reports included histological subtype, Lauren classification, pathological T stage, pathological nodal stage, number of metastatic lymph nodes, total number of examined lymph nodes, lymph node ratio (LNR), and tumor regression grade (TRG). Tumor regression grade was assessed using the CAP tumor regression grading system. For descriptive analyses, TRG was categorized as any pathologic regression present (TRG 0–2) or poor/no pathologic regression (TRG 3).

### Perioperative chemotherapy (FLOT regimen)

Patients received perioperative chemotherapy with the FLOT regimen every 2 weeks as follows:

• **Oxaliplatin** 85 mg/m^2^, 2-hour infusion
• **Docetaxel** 50 mg/m^2^, 1-hour infusion
• **Folinic acid (Leucovorin)** 200 mg/m^2^, 2-hour infusion
• **5-Fluorouracil** 2,600 mg/m^2^**,** 24-hour continuous infusion

### Systemic Immune-Inflammation Index and Lymph Node Ratio calculations

• Systemic Immune-Inflammation Index

SII was calculated from pretreatment complete blood count parameters using neutrophil, platelet, and lymphocyte values.

\begin{eqnarray*}SII= \frac{Neutrophil~count \left( \times \frac{1{0}^{9}}{L} \right) \times Platelet~count(\times 1{0}^{9}/L)}{Lymphocyte~count(\times 1{0}^{9}/L)} \end{eqnarray*}

• Lymph Node Ratio

LNR was calculated from postoperative pathology reports by dividing the number of metastatic lymph nodes by the total number of examined lymph nodes.

\begin{eqnarray*}LNR= \frac{Number~of~metastatic~lymph~nodes~(n)}{Total~number~of~lymph~nodes~removed~(n)} . \end{eqnarray*}

### Defining the combined risk score

In this study, the SII and LNR were combined to develop a composite risk scoring system with three categories for postoperative outcome stratification. Cut-off values for SII and LNR were determined using receiver operating characteristic (ROC) curve analysis. Patients were then stratified into low-, moderate-, and high-risk groups according to their SII and LNR categories, and the combined SII–LNR risk score was constructed accordingly.

### Follow-up and outcome assessment

After completion of perioperative treatment and surgery, patients were followed according to institutional routine clinical practice. Because this was a retrospective real-world study, follow-up intervals and surveillance procedures were not fully standardized across all patients. Follow-up information was obtained from the institutional electronic health record system and patient files. Recurrence was determined based on radiological, pathological, or clinical assessment documented in the medical records. Overall survival (OS) was defined as the time from diagnosis to death from any cause, and disease-free survival (DFS) as the time from diagnosis to recurrence or death, whichever occurred first.

### Statistical analysis

Statistical analyses were performed using SPSS (version 21; IBM Corp., Armonk, NY, USA) and jamovi (version 2.6.17). Normality was assessed using the Shapiro–Wilk test. Survival curves were estimated using the Kaplan–Meier method and compared with the log-rank test. Median follow-up time was estimated using the reverse Kaplan–Meier method. Multivariable analyses were performed using Cox proportional hazards regression. ROC curve analysis was performed using 5-year overall survival (OS) status as the state variable, and the optimal cut-off values for SII and LNR were determined using Youden’s J index. The ROC-derived cut-off values based on 5-year OS status were subsequently applied to both OS and disease-free survival (DFS) analyses in order to maintain a single predefined combined risk classification. Patients with missing or incomplete required clinical, laboratory, and pathological data were excluded from the final analytic cohort; therefore, complete-case analysis was used. Patients who did not complete perioperative FLOT were also excluded from the study. However, relative dose intensity (RDI) was not analyzed because dose reductions and treatment delays were not captured in a sufficiently standardized manner for reliable retrospective calculation. A two-sided *p* value <0.05 was considered statistically significant.

### Ethics committee approval

The study protocol was reviewed and approved by the Ethics Committee of Adana City Training and Research Hospital (Meeting No: 15, Date: July 10, 2025, Approval No: 625). Institutional permission for data use was obtained from the hospital administration. The requirement for written informed consent was waived by the Ethics Committee due to the retrospective design and the use of fully anonymized data.

## Results

The final study cohort included 153 patients. The median age was 64 years (IQR, 57–71), and 111 patients (72.5%) were male. According to the institutional records, 118 patients had gastric adenocarcinoma and 35 had GEJ adenocarcinoma. Tumor location was recorded as upper third in 62 patients (40.5%), middle third in 32 (20.9%), and lower third in 59 (38.6%). Total gastrectomy was performed in 125 patients (81.7%), whereas 28 patients (18.3%) underwent subtotal gastrectomy. Pathological T stage was T2 in 49 patients (32.0%), T3 in 67 (43.8%), and T4 in 37 (24.2%). Nodal status was N0 in 56 patients (36.6%), N1 in 35 (22.9%), N2 in 32 (20.9%), and N3 in 30 (19.6%). Histologically, 111 tumors (72.5%) were adenocarcinoma, 38 (24.8%) signet-ring cell carcinoma, and four (2.6%) mucinous carcinoma. Lauren classification was intestinal in 93 patients (60.8%) and diffuse in 60 (39.2%). TRG was categorized as any pathologic regression present (TRG 0–2) or poor/no pathologic regression (TRG 3). Overall, any pathologic regression was present in 61 patients (39.9%), whereas poor/no pathologic regression was observed in 92 patients (60.1%). Detailed baseline characteristics across the combined risk groups are presented in Table 1. The median follow-up time was 47.3 months. During follow-up, 98 patients died. In the DFS analysis, recurrence or death was documented in 109 patients.

**Table 1:** Comparisons of demographics and clinical characteristics among combined risk score groups.

		**Combined risk score groups**			
**Variable**	**Overall (*n* = 153)**	**Low-risk (*n* = 50)**	**Moderate-risk (*n* = 67)**	**High-risk (*n* = 36)**	***p***
**Age, median (IQR), years**	64 (57–71)	64 (58–70)	63 (56–72)	64 (54–72)	0.779
**Sex**					
Female	42 (27.5)	19 (38.0)	13 (19.4)	10 (27.8)	0.083
Male	111 (72.5)	31 (62.0)	54 (80.6)	26 (72.2)	
**Surgery type**					
Total	125 (81.7)	39 (78.0)	54 (80.6)	32 (88.9)	0.415
Subtotal	28 (18.3)	11 (22.0)	13 (19.4)	4 (11.1)	
**T stage**					
T2	49 (32.0)	20 (40.0)	22 (32.8)	7 (19.4)	0.242
T3	67 (43.8)	22 (44.0)	27 (40.3)	18 (50.0)	
T4	37 (24.2)	8 (16.0)	18 (26.9)	11 (30.6)	
**Tumor location**					
Lower	59 (38.6)	15 (30.0)	27 (40.3)	17 (47.2)	0.357
Middle	32 (20.9)	13 (26.0)	15 (22.4)	4 (11.1)	
Upper	62 (40.5)	22 (44.0)	25 (37.3)	15 (41.7)	
**Histopathology**					
Adenocarcinoma	111 (72.5)	42 (84.0)	51 (76.1)	18 (50.0)	0.004
Signet-ring cell carcinoma	38 (24.8)	8 (16.0)	15 (22.4)	15 (41.7)	
Mucinous carcinoma	4 (2.6)	0 (0.0)	1 (1.5)	3 (8.3)	
**Lauren**					
Diffuse	60 (39.2)	10 (20.0)	26 (38.8)	24 (66.7)	0.001
Intestinal	93 (60.8)	40 (80.0)	41 (61.2)	12 (33.3)	
**Pathologic regression**					
Poor/no regression (TRG 3)	92 (60.1)	20 (40.0)	44 (65.7)	28 (77.8)	<0.001
Any regression present (TRG 0–2)	61 (39.9)	30 (60.0)	23 (34.3)	8 (22.2)	

### ROC analysis

**Figure 2:**
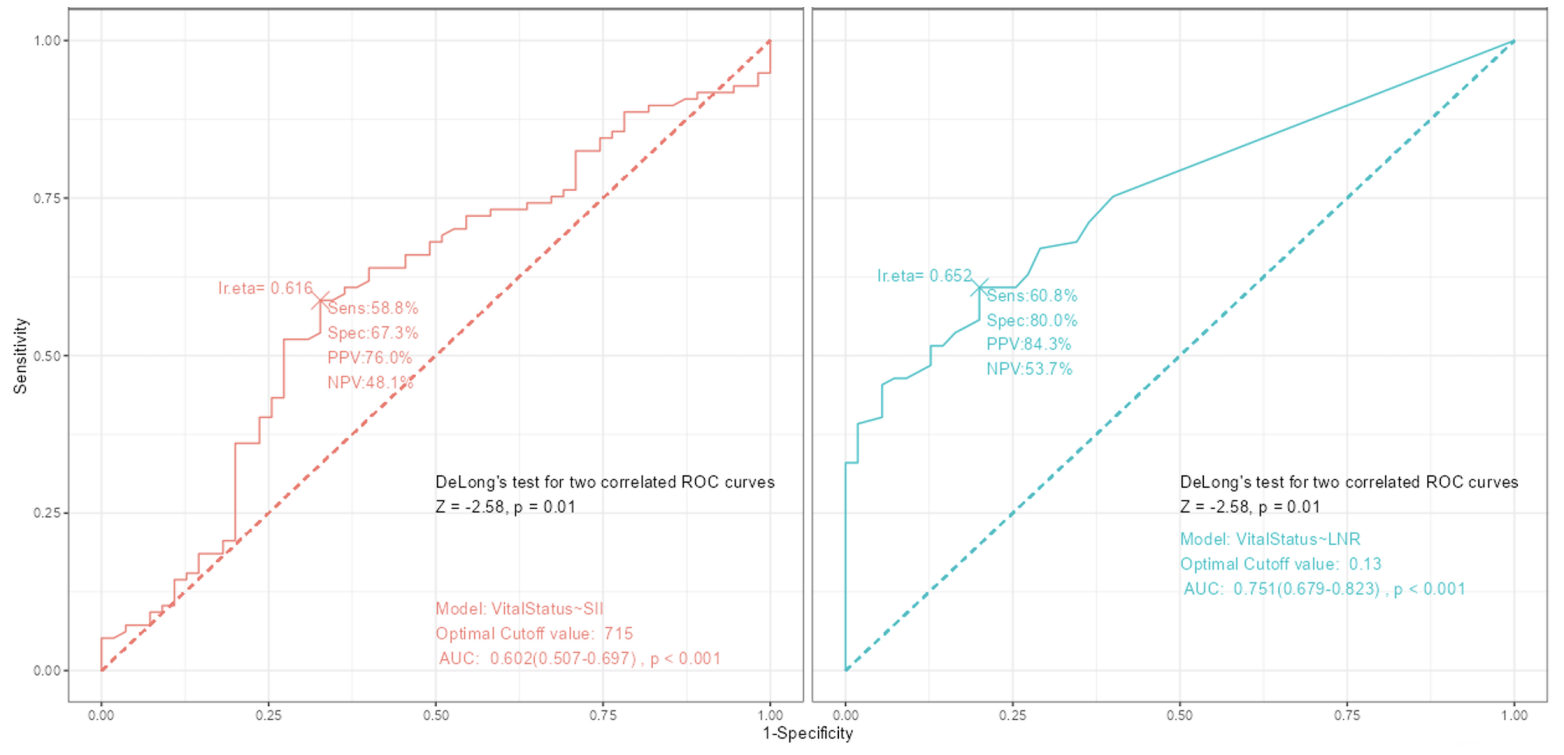
ROC curves.

Receiver operating characteristic (ROC) analysis was performed using 5-year overall survival status as the state variable. The AUC was 0.602 for SII and 0.751 for LNR. Using Youden’s J index, the optimal cut-off values were 715 for SII and 0.13 for LNR; values above these thresholds were considered adverse (Fig. 2).

### Combined SII–LNR risk score

In this study, the SII and LNR were used to create a combined risk scoring system with three risk categories for postoperative outcome stratification. Cut-off values determined for SII and LNR were 715 and 0.13, respectively. Accordingly, the low-, moderate- and high-risk groups were as follows:

• **Low-risk group**: Patients with a SII value of ≤715 and a LNR value of ≤0.13
• **Moderate-risk group**: Patients with a SII value of >715 and a LNR value of ≤0.13 or a SII value of ≤715 and a LNR value of >0.13
• **High-risk group**: Patients with a SII value of >715 and a LNR value of >0.13

The combined SII–LNR score categorized patients into low-risk (*n* = 50, 32.7%), moderate-risk (*n* = 67, 43.8%), and high-risk (*n* = 36, 23.5%) groups.

No statistically significant differences were observed among the low-, moderate-, and high-risk groups in age, sex distribution, surgery type, T stage, or tumor location. The rates of signet-ring cell and mucinous histology were significantly higher in the high-risk group. Pathologic regression rates varied across the risk groups, with any pathologic regression observed in 60.0% of patients in the low-risk group, 34.3% in the moderate-risk group, and 22.2% in the high-risk group (Table 1).

**Figure 3:**
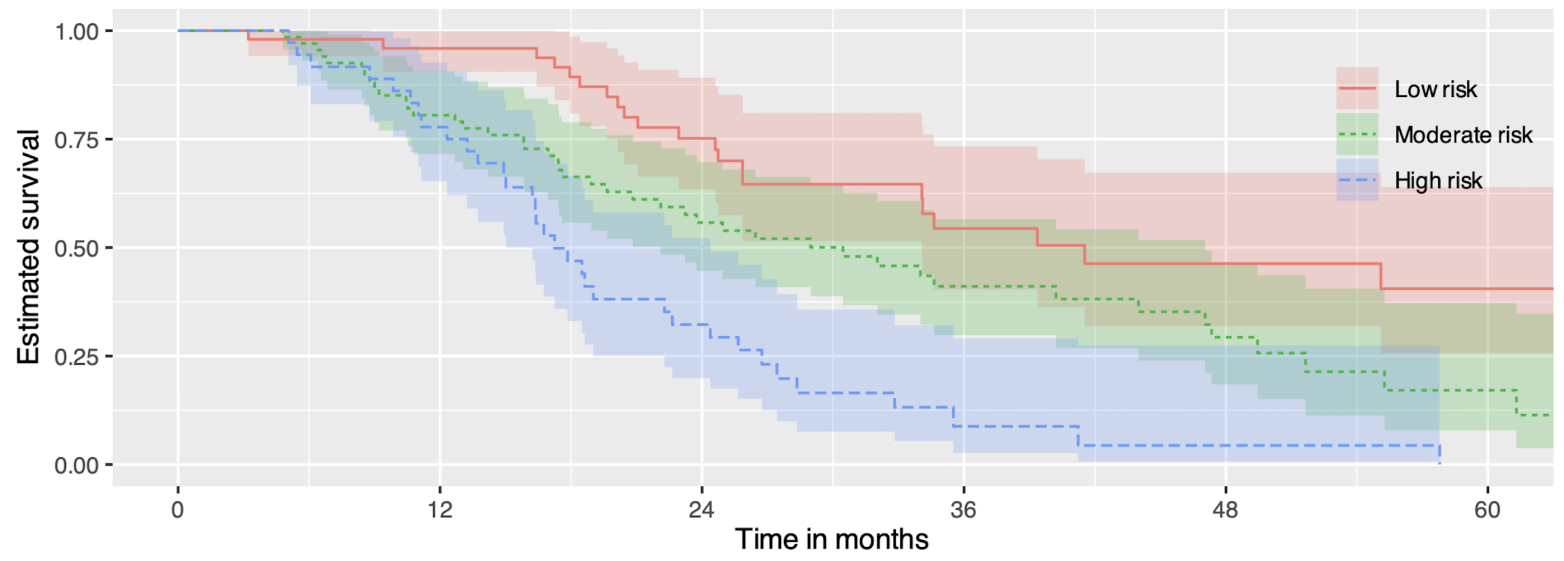
Kaplan–Meier overall survival by combined risk groups.

The median overall survival for the entire cohort was 26.4 months. In the Kaplan–Meier analysis by combined risk groups, median overall survival was 41.5 months (95% CI [20.5–62.5]) in the low-risk group, 30.5 months (95% CI [19.7–41.2]) in the moderate-risk group, and 17.2 months (95% CI [14.2–20.2]) in the high-risk group. The log-rank test demonstrated statistically significant differences in overall survival among the risk groups (*p* < 0.001, Fig. 3).

The 5-year overall survival (OS) rates in the low-, moderate-, and high-risk groups were 40.5%, 17.1%, and 4.4%, respectively. In multivariable Cox regression analysis, compared with the low-risk group, the risk of death was significantly higher in the moderate-risk group (HR 1.93; 95% CI [1.15–3.26]; *p* = 0.013) and the high-risk group (HR 4.13; 95% CI [2.37–7.22]; *p* < 0.001).

In a separate multivariable Cox regression model evaluating individual clinicopathological variables, age, middle-third tumor location, and LNR were independently associated with overall survival. Each one-year increase in age was associated with a higher risk of death (HR 1.03; 95% CI [1.01–1.06]; *p* = 0.005). Compared with lower-third tumors, middle-third tumor location was associated with higher mortality (HR 1.91; 95% CI [1.05–3.47]; *p* = 0.035), whereas upper-third tumor location was not significantly associated with mortality (HR 1.19; 95% CI [0.68–2.07]; *p* = 0.540). In addition, each 0.1 increase in LNR was associated with an increased risk of death (HR 1.45; 95% CI [1.23–1.71]; *p* < 0.001) (Table 2).

**Table 2:** Mortality estimates from multivariate survival analysis.

		**All, n (%)**	**Univariable HR (95% CI, *p*)**	**Multivariable HR (95% CI, *p*)**
Sex	Male	111 (72.5)	-	-
	Female	42 (27.5)	1.12 (0.72–1.76, *p* = 0.606)	1.41 (0.84–2.36, *p* = 0.198)
Surgery type	Total gastrectomy	125 (81.7)	-	-
	Subtotal gastrectomy	28 (18.3)	0.84 (0.50–1.42, *p* = 0.514)	1.09 (0.56–2.14, *p* = 0.801)
Histological subtype	Adenocarcinoma	111 (72.5)	-	-
	Signet-ring cell carcinoma	38 (24.8)	1.79 (1.15–2.79, *p* = 0.010)	0.91 (0.44–1.92, *p* = 0.813)
	Mucinous carcinoma	4 (2.6)	10.64 (3.62–31.30, *p* < 0.001)	2.05 (0.53–7.93, *p* = 0.297)
T Stage	T2	49 (32.0)	-	-
	T3	67 (43.8)	1.46 (0.88–2.41, *p* = 0.140)	0.84 (0.48–1.47, *p* = 0.538)
	T4	37 (24.2)	2.54 (1.46–4.41, *p* = 0.001)	1.34 (0.67–2.67, *p* = 0.406)
Tumor location	Lower third	59 (38.6)	-	-
	Middle third	32 (20.9)	1.47 (0.87–2.48, *p* = 0.152)	1.91 (1.05–3.47, *p* = 0.035)
	Upper third	62 (40.5)	1.02 (0.64–1.61, *p* = 0.943)	1.19 (0.68–2.07, *p* = 0.540)
Lauren classification	Diffuse	60 (39.2)	-	-
	Intestinal	93 (60.8)	0.56 (0.38–0.84, *p* = 0.005)	1.25 (0.56–2.78, *p* = 0.583)
Grade	Grade 1	19 (12.4)	-	-
	Grade 2	52 (34.0)	1.59 (0.66–3.81, *p* = 0.301)	1.26 (0.51–3.15, *p* = 0.617)
	Grade 3	82 (53.6)	2.86 (1.23–6.62, *p* = 0.014)	1.84 (0.67–5.04, *p* = 0.233)
LNR (per 0.1 increase)	Mean (SD)	0.2 (0.2)	1.37 (1.26–1.49, *p* < 0.001)	1.45 (1.23–1.71, *p* < 0.001)
Age	Median (IQR)	64 (57–71)	1.00 (0.98–1.02, *p* = 0.818)	1.03 (1.01–1.06, *p* = 0.005)

Patients with N3 disease comprised 19.6% of the cohort (30/153). In univariable analysis, N3 status was associated with worse mortality compared with N0 disease. However, this association was not retained as an independent prognostic factor after adjustment in the multivariable model, suggesting that its effect was partly captured by other postoperative disease-burden variables, particularly LNR.

**Figure 4:**
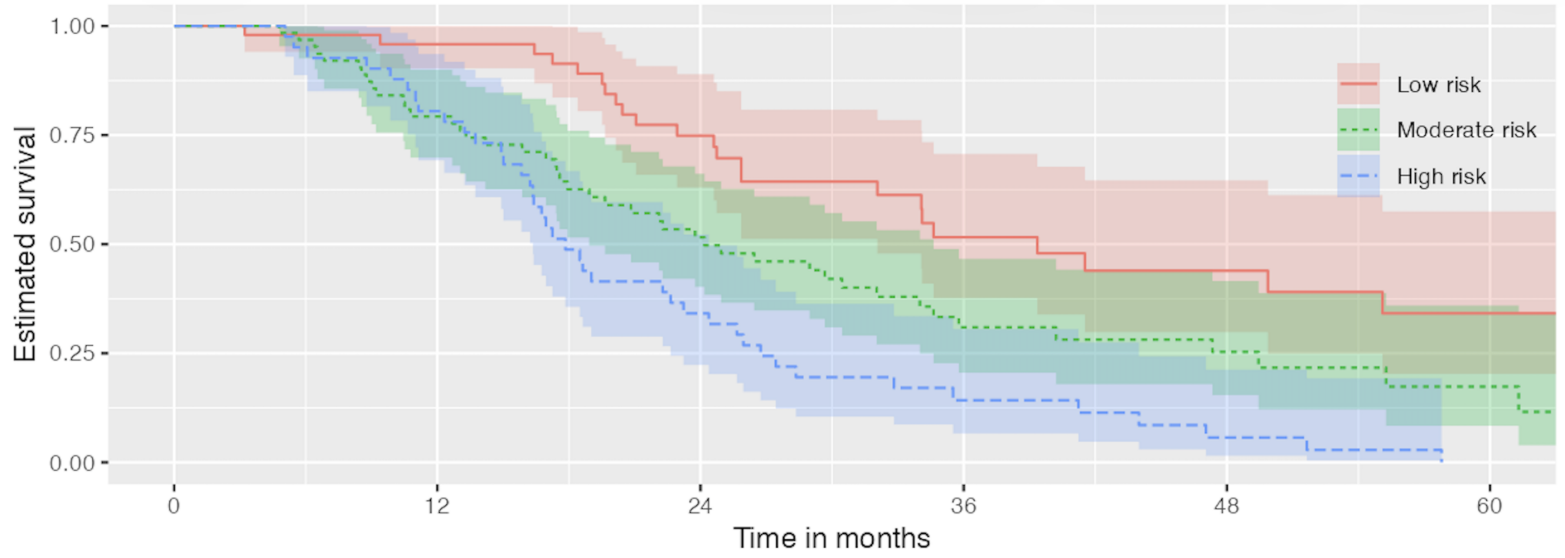
Kaplan–Meier disease-free survival by combined risk groups.

The analysis of DFS by combined risk group showed that median DFS was 32.4 months in the low-risk group, 15.6 months in the moderate-risk group, and 10.8 months in the high-risk group (log-rank *p* < 0.001; Fig. 4). The 5-year DFS rates were 34.2%, 17.4%, and 5.7%, respectively. In the multivariable Cox regression analysis, the risk of recurrence or death was significantly higher in the moderate-risk group (HR 2.07; 95% CI [1.25–3.42]; *p* = 0.005) and in the high-risk group (HR 3.58; 95% CI [2.12–6.02]; *p* < 0.001) compared with the low-risk group.

As an exploratory descriptive analysis, the moderate-risk group was further separated into two subgroups: patients with elevated SII only and those with elevated LNR only. Among the moderate-risk patients, 38 had high SII only and 29 had high LNR only. Median overall survival was 34.0 months in the high-SII-only subgroup and 20.8 months in the high-LNR-only subgroup. Median disease-free survival was 22.6 months and 12.4 months, respectively. These findings suggest heterogeneity within the moderate-risk category. Numerically, survival outcomes appeared poorer in patients with isolated LNR elevation than in those with isolated SII elevation; however, this subgroup analysis was exploratory and descriptive.

## Discussion

Despite advances in multimodal treatment, gastric and gastroesophageal junction adenocarcinomas remain associated with substantial morbidity and mortality (Sung et al., 2021). Perioperative systemic therapy is intended to treat micrometastatic disease, reduce primary tumor and nodal burden, improve the likelihood of curative resection, and promote pathological tumor regression (Ychou et al., 2011). Postoperative outcomes nevertheless remain heterogeneous, even among patients treated with contemporary perioperative regimens.

In the present real-world cohort of 153 resected patients treated with perioperative FLOT, the median overall survival for the entire cohort was 26.4 months. The median OS reported in the FLOT4-AIO trial was longer than that observed in our cohort (Al-Batran et al., 2019). This difference may reflect the retrospective single-center design of the present study and the fact that real-world populations often include more comorbidities, more variable treatment adherence, and greater perioperative heterogeneity than trial populations. In addition, the treatment landscape is changing. The phase 3 MATTERHORN trial showed improved event-free survival with perioperative durvalumab plus FLOT compared with placebo plus FLOT (Janjigian et al., 2025). Accordingly, the applicability of our findings to patients receiving perioperative chemoimmunotherapy remains uncertain.

The SII, derived from platelet, neutrophil, and lymphocyte counts, has been shown to have significant prognostic value in various malignancies including lung cancer, colorectal cancer and hepatocellular carcinoma (Zhang et al., 2019; Chen et al., 2017; Wang, Huang & Lin, 2020). Wang et al. (2017) reported that higher preoperative SII levels were associated with shorter OS (65 months *vs.* 22 months) and 5-year overall survival rates were 46.1% in the lower SII group and 29.4% in the higher SII group in a study conducted in 444 patients with gastric cancer. Likewise, prior data suggest that the lymph node ratio (LNR), defined as the ratio of metastatic to examined lymph nodes, may also serve as a useful prognostic marker in patients with locally advanced gastric cancer receiving neoadjuvant chemotherapy (Zhu et al., 2021; Jiang et al., 2022). In a study conducted by Jannasch et al. (2025) 5-year survival rates by LNR were reported as 43.8% in the LNR 0.1–0.2 group and 12.2% in the LNR 0.41–1 group, indicating a significant reduction in overall survival as the LNR gradually increased.

Available data suggest that combining inflammatory and nutritional indices may improve prognostic discrimination. Ding et al. (2024) evaluated an SII–PNI scoring system combining the SII and the PNI, in 181 patients with locally advanced gastric cancer receiving neoadjuvant chemotherapy. In this scoring system, a score of 2 is given for both high SII and low PNI, a score of 1 is given for either high SII or low PNI alone and a score of 0 is given if both high SII and low PNI are absent. In that study, five-year OS and DFS rates were reported as 21% and 15% in patients with high SII–PNI score whereas these rates were 59% and 53% in patients with a SII–PNI score of 0; furthermore, a multivariate analysis showed that SII–PNI was an independent prognostic factor. Similarly, Li, Zhang & Fu (2024) reported that the ICPI scoring system derived from neutrophil-to-lymphocyte ratio (NLR), platelet-to-lymphocyte ratio (PLR) and monocyte-to-lymphocyte ratio (MLR) was superior in predicting survival compared to these parameters alone.

However, there are no prior studies that have comprehensively assessed SII and LNR as a single combined risk score in patients with gastric and GEJ cancer treated with perioperative FLOT. In the present study, the combined SII–LNR score stratified patients into distinct postoperative OS and DFS risk groups. Kaplan–Meier analyses demonstrated significant survival differences among the low-, moderate-, and high-risk categories, and Cox regression analysis showed progressively higher risks of death and recurrence across these groups. These findings suggest that the combination of pretreatment SII and postoperative LNR may be useful for postoperative risk stratification in routine practice.

SII and LNR likely reflect different biological aspects of the disease. SII represents the systemic inflammatory and immune milieu before treatment, whereas LNR reflects postoperative nodal tumor burden after pathological assessment. This separation may reflect the combined effect of pretreatment systemic inflammation and postoperative nodal disease burden. A strength of the present study is that it was conducted in a real-world cohort of patients treated with perioperative FLOT using routinely available laboratory and pathological parameters. However, the study should be interpreted as evaluating a postoperative prognostic tool rather than a predictive model for treatment selection. When interpreting the multivariable analyses, some modeling considerations should also be acknowledged. Although pathologic response is clinically relevant, TRG was not included in the primary multivariable model because of the limited number of events and the risk of model overfitting; therefore, it was presented descriptively rather than treated as a primary adjustment variable.

This study has several limitations. Its retrospective and single-center design may have introduced selection bias and limits the generalizability of the findings. In addition, because the final analytic cohort included only patients who underwent surgery and completed perioperative FLOT, selection bias toward patients with better treatment tolerance cannot be excluded. The relatively limited sample size may also have reduced the statistical power of subgroup analyses. Although the cohort included both gastric and gastroesophageal junction adenocarcinomas, separate site-specific survival analyses were not performed because of the retrospective study design and limited subgroup sizes. In addition, treatment exposure, including dose reductions and treatment delays, and follow-up duration were not fully uniform across all patients, which may have influenced survival outcomes. Therefore, the proposed SII–LNR score requires external validation in larger, prospective, multicenter cohorts before it can be incorporated into routine clinical decision-making.

## Conclusions

In this study, the combined SII–LNR score stratified patients with resectable gastric and gastroesophageal junction adenocarcinoma treated with perioperative FLOT into distinct postoperative risk groups for overall survival and disease-free survival. As a simple score derived from routine laboratory and pathological data, it may help identify patients who warrant closer postoperative surveillance and risk counseling. However, these findings require validation in independent multicenter cohorts.

## Supplemental Information

10.7717/peerj.21499/supp-1 Supplemental Information 1 Dataset (De-identified raw measurements).

10.7717/peerj.21499/supp-2 Supplemental Information 2 Codebook.

10.7717/peerj.21499/supp-3 Supplemental Information 3 STROBE Checklist.

## Additional Information and Declarations

### Data Availability

The following information was supplied regarding data availability: The raw measurements are available in the Supplemental Files.
