# Supplementary material for: Development and prognostic evaluation of a combined SII–LNR score in resectable gastric and gastroesophageal junction adenocarcinoma treated with perioperative FLOT: a retrospective single-center study
Source: PeerJ. 2026 Jun 29;14:e21499. doi: 10.7717/peerj.21499 (PMC13326649; doi:10.7717/peerj.21499)
Supplement: Supplemental Information 2 [file peerj-14-21499-s002.docx]

# CODEBOOK

| **Variable name** | **Type** | **Coding / Values** | **Description** |
| --- | --- | --- | --- |
| Sex | binary categorical | Male; Female (text categories) | Patient sex as recorded in the medical chart. |
| Age | continuous | Numeric value in years | Age at diagnosis. |
| Surgery type | categorical | Total gastrectomy; Subtotal gastrectomy | Type of gastrectomy performed. |
| Histological subtype | categorical | Adenocarcinoma; Mucinous Carcinoma; Signet ring cell carcinoma | Histological subtype of the primary tumor. |
| T stage | ordinal categorical | T2; T3; T4 | Pathological T stage (TNM). Higher categories indicate more advanced local tumor invasion. |
| Nodal Status | ordinal categorical | N0; N1; N2; N3 | Pathological N stage (TNM). |
| Tumour localisation | categorical | Lower third; Middle third; Upper third | Anatomical location of the primary tumor in the stomach. |
| Response status | binary categorical | Present; Absent | Radiologic/clinical response to perioperative FLOT as defined in the study. |
| TRG | ordinal categorical (numeric) | 0, 1, 2, 3 (numeric categories; lower scores indicate better histologic tumor regression and higher scores indicate poorer regression) | Tumor regression grade on surgical specimens, coded from 0 to 3 according to the histopathological tumor regression grading system used in the pathology department. |
| Date of Diagnosis | date | Calendar date (YYYY-MM-DD) | Date of initial diagnosis of gastric or gastroesophageal junction adenocarcinoma. |
| Date of Disease Recurrence | date | Calendar date (YYYY-MM-DD) | Date of recurrence. For patients without documented recurrence at last follow-up, this date was set equal to the last clinical visit date (censored). |
| Date of Death | date | Calendar date (YYYY-MM-DD) | Date of death. For patients who were alive at last follow-up, this date was set equal to the last clinical visit date (censored). |
| Platelet | continuous | Numeric value | Platelet count from peripheral blood at the time point used in the study (as recorded in the laboratory system). |
| Neutrophil | continuous | Numeric value | Neutrophil count from peripheral blood at the time point used in the study (as recorded in the laboratory system). |
| Lymphocyte | continuous | Numeric value | Lymphocyte count from peripheral blood at the time point used in the study (as recorded in the laboratory system). |
| SII | continuous (derived index) | Numeric value | Systemic immune-inflammation index (SII), calculated from platelet, neutrophil, and lymphocyte counts according to the study protocol. |
| ALBUMIN | continuous | Numeric value | Serum albumin level at the time point used in the study. |
| PNI | continuous (derived index) | Numeric value | Prognostic Nutritional Index (PNI), derived from serum albumin and lymphocyte count according to the study protocol. |
| Number of Positive Lymph Nodes | discrete numeric | Non-negative integer | Number of metastatic (positive) lymph nodes in the surgical specimen. |
| Total Number of Lymph Nodes | discrete numeric | Positive integer | Total number of examined lymph nodes in the surgical specimen. |
| LNR | continuous (ratio) | Numeric value between 0 and 1 | Lymph node ratio, calculated as the number of positive lymph nodes divided by the total number of examined lymph nodes. |
| Lauren classification | categorical | Diffuse; Intestinal | Histologic subtype according to Lauren classification. |
| Grade | ordinal categorical (numeric) | 1 = well differentiated; 2 = moderately differentiated; 3 = poorly differentiated | Histologic tumor grade. |
| Recurrence Status | binary categorical (numeric) | 0 = no documented recurrence at last visit (censored); 1 = disease recurrence occurred | Recurrence event indicator used for disease-free survival analyses. For patients without documented recurrence, the Date of Disease Recurrence was set to the last clinical visit date and the event was coded as 0 (censored). |
| Vital Status | binary categorical (numeric) | 0 = alive at last visit (censored); 1 = death occurred | Overall survival event indicator. For patients who were alive at last follow-up, the Date of Death was set to the last clinical visit date and the event was coded as 0 (censored). |
