# Supplementary material for: Development and prognostic evaluation of a combined SII–LNR score in resectable gastric and gastroesophageal junction adenocarcinoma treated with perioperative FLOT: a retrospective single-center study
Source: PeerJ. 2026 Jun 29;14:e21499. doi: 10.7717/peerj.21499 (PMC13326649; doi:10.7717/peerj.21499)
Supplement: Supplemental Information 3 [file peerj-14-21499-s003.doc]

**Development and Prognostic Evaluation of a Combined SII–LNR Score in Resectable Gastric and Gastroesophageal Junction Adenocarcinoma Treated with Perioperative FLOT: A Retrospective Single-Center Study**

|  | Item No | Recommendation |
| --- | --- | --- |
| **Title and abstract** | 1 | (*a*) Indicate the study’s design with a commonly used term in the title or the abstract  **Location in manuscript:** Title; Abstract – Methods |
|  |  | (*b*) Provide in the abstract an informative and balanced summary of what was done and what was found  **Location in manuscript:** Abstract – Background, Methods, Results, and Conclusion |
| Introduction | | |
| Background/rationale | 2 | Explain the scientific background and rationale for the investigation being reported  **Location in manuscript:** Introduction |
| Objectives | 3 | State specific objectives, including any prespecified hypotheses  **Location in manuscript:** Introduction – final paragraph |
| Methods | | |
| Study design | 4 | Present key elements of study design early in the paper  **Location in manuscript:** Materials and Methods – Study Design and Site |
| Setting | 5 | Describe the setting, locations, and relevant dates, including periods of recruitment, exposure, follow-up, and data collection  **Location in manuscript:** Materials and Methods – Study Design and Site; Patient Selection and Study Population |
| Participants | 6 | (*a*) Give the eligibility criteria, and the sources and methods of selection of participants. Describe methods of follow-up  **Location in manuscript:** Materials and Methods – Patient Selection and Study Population; Inclusion Criteria; Exclusion Criteria; Follow-up and Outcome Assessment |
|  |  | (*b*) For matched studies, give matching criteria and number of exposed and unexposed  **Location in manuscript:** Not applicable; this was not a matched study. |
| Variables | 7 | Clearly define all outcomes, exposures, predictors, potential confounders, and effect modifiers. Give diagnostic criteria, if applicable  **Location in manuscript:** Materials and Methods – Clinical and Demographic Data Collection; Histopathological Assessment; SII and LNR Calculations; Defining the Combined Risk Score; Follow-up and Outcome Assessment |
| Data sources/ measurement | 8* | For each variable of interest, give sources of data and details of methods of assessment (measurement). Describe comparability of assessment methods if there is more than one group  **Location in manuscript:** Materials and Methods – Clinical and Demographic Data Collection; Histopathological Assessment; SII and LNR Calculations; Follow-up and Outcome Assessment |
| Bias | 9 | Describe any efforts to address potential sources of bias  **Location in manuscript:** Materials and Methods – Patient Selection and Study Population; Exclusion Criteria; Statistical Analysis; Discussion – Limitations |
| Study size | 10 | Explain how the study size was arrived at  **Location in manuscript:** Materials and Methods – Patient Selection and Study Population; Results – first paragraph; Figure 1 |
| Quantitative variables | 11 | Explain how quantitative variables were handled in the analyses. If applicable, describe which groupings were chosen and why  **Location in manuscript:** Materials and Methods – SII and LNR Calculations; Defining the Combined Risk Score; Statistical Analysis |
| Statistical methods | 12 | (*a*) Describe all statistical methods, including those used to control for confounding  **Location in manuscript:** Materials and Methods – Statistical Analysis |
|  |  | (*b*) Describe any methods used to examine subgroups and interactions  **Location in manuscript:** Results – exploratory descriptive analysis of the moderate-risk group; Materials and Methods – Statistical Analysis |
|  |  | (*c*) Explain how missing data were addressed  **Location in manuscript:** Materials and Methods – Statistical Analysis |
|  |  | (*d*) If applicable, explain how loss to follow-up was addressed  **Location in manuscript:** Materials and Methods – Follow-up and Outcome Assessment; Statistical Analysis |
|  |  | (*e*) Describe any sensitivity analyses  **Location in manuscript:** Not applicable; no sensitivity analyses were performed. |
| Results | | |
| Participants | 13* | (a) Report numbers of individuals at each stage of study—eg numbers potentially eligible, examined for eligibility, confirmed eligible, included in the study, completing follow-up, and analysed  **Location in manuscript:** Materials and Methods – Patient Selection and Study Population; Results – first paragraph; Figure 1 |
|  |  | (b) Give reasons for non-participation at each stage  **Location in manuscript:** Materials and Methods – Patient Selection and Study Population; Exclusion Criteria; Figure 1 |
|  |  | (c) Consider use of a flow diagram  **Location in manuscript:** Figure 1 |
| Descriptive data | 14* | (a) Give characteristics of study participants (eg demographic, clinical, social) and information on exposures and potential confounders  **Location in manuscript:** Results – first paragraph; Table 1 |
|  |  | (b) Indicate number of participants with missing data for each variable of interest  **Location in manuscript:** Materials and Methods – Patient Selection and Study Population; Exclusion Criteria; Statistical Analysis |
|  |  | (c) Summarise follow-up time (eg, average and total amount)  **Location in manuscript:** Results – first paragraph |
| Outcome data | 15* | Report numbers of outcome events or summary measures over time  **Location in manuscript:** Results – first paragraph; Results – Overall Survival and Disease-Free Survival analyses |
| Main results | 16 | (*a*) Give unadjusted estimates and, if applicable, confounder-adjusted estimates and their precision (eg, 95% confidence interval). Make clear which confounders were adjusted for and why they were included  **Location in manuscript:** Results – Combined SII–LNR Risk Score; Table 2; multivariable Cox regression analyses |
|  |  | (*b*) Report category boundaries when continuous variables were categorized  **Location in manuscript:** Materials and Methods – Defining the Combined Risk Score; Results – ROC Analysis; Results – Combined SII–LNR Risk Score |
|  |  | (*c*) If relevant, consider translating estimates of relative risk into absolute risk for a meaningful time period  **Location in manuscript:** Results – Combined SII–LNR Risk Score; 5-year OS and DFS rates |
| Other analyses | 17 | Report other analyses done—eg analyses of subgroups and interactions, and sensitivity analyses  **Location in manuscript:** Results – exploratory descriptive analysis of the moderate-risk group |
| Discussion | | |
| Key results | 18 | Summarise key results with reference to study objectives  **Location in manuscript:** Discussion – first paragraph and main findings paragraphs |
| Limitations | 19 | Discuss limitations of the study, taking into account sources of potential bias or imprecision. Discuss both direction and magnitude of any potential bias  **Location in manuscript:** Discussion – Limitations paragraph |
| Interpretation | 20 | Give a cautious overall interpretation of results considering objectives, limitations, multiplicity of analyses, results from similar studies, and other relevant evidence  **Location in manuscript:** Discussion; Conclusions |
| Generalisability | 21 | Discuss the generalisability (external validity) of the study results  **Location in manuscript:** Discussion – Limitations paragraph; Conclusions |
| Other information | | |
| Funding | 22 | Give the source of funding and the role of the funders for the present study and, if applicable, for the original study on which the present article is based  **Location in manuscript:** Funding |
